# Comparison of Different Strategies for Selection/Adaptation of Mixed Microbial Cultures Able to Ferment Crude Glycerol Derived from Second-Generation Biodiesel

**DOI:** 10.1155/2015/932934

**Published:** 2015-10-05

**Authors:** C. Varrone, T. M. B. Heggeset, S. B. Le, T. Haugen, S. Markussen, I. V. Skiadas, H. N. Gavala

**Affiliations:** ^1^Department of Chemistry and Biosciences, Aalborg University, 2350 Copenhagen, Denmark; ^2^Department of Chemical and Biochemical Engineering, Technical University of Denmark, 2800 Lyngby, Denmark; ^3^Biotechnology and Nanomedicine, SINTEF Materials and Chemistry, 7465 Trondheim, Norway

## Abstract

Objective of this study was the selection and adaptation of mixed microbial cultures (MMCs), able to ferment crude glycerol generated from animal fat-based biodiesel and produce building-blocks and green chemicals. Various adaptation strategies have been investigated for the enrichment of suitable and stable MMC, trying to overcome inhibition problems and enhance substrate degradation efficiency, as well as generation of soluble fermentation products. Repeated transfers in small batches and fed-batch conditions have been applied, comparing the use of different inoculum, growth media, and Kinetic Control. The adaptation of activated sludge inoculum was performed successfully and continued unhindered for several months. The best results showed a substrate degradation efficiency of almost 100% (about 10 g/L glycerol in 21 h) and different dominant metabolic products were obtained, depending on the selection strategy (mainly 1,3-propanediol, ethanol, or butyrate). On the other hand, anaerobic sludge exhibited inactivation after a few transfers. To circumvent this problem, fed-batch mode was used as an alternative adaptation strategy, which led to effective substrate degradation and high 1,3-propanediol and butyrate production. Changes in microbial composition were monitored by means of Next Generation Sequencing, revealing a dominance of glycerol consuming species, such as* Clostridium*,* Klebsiella*, and* Escherichia*.

## 1. Introduction

The exponential growth of biodiesel production in the last decade has led to a concomitant increase in crude glycerol [1, 2]. Hence, new uses of crude glycerol are required in order to overcome the problem of glycerol glut. Methods for glycerol utilization or disposal include combustion, composting, anaerobic digestion, animal feed, and thermochemical or biological conversion to value-added products [3]. New methods for the valorization of glycerol involve the bioconversion into biofuels and green chemicals, which might provide several advantages, compared to some of the above-mentioned methods. Environmental biotechnologies are thus going to provide a significant contribution to tackle the challenge of a more efficient use of by-products and waste streams. In this frame, a so-called “ecobiotechnological approach” has been recently proposed as an interesting tool for a more effective exploitation of wastes and wastewaters [4].

As stated by Johnson and colleagues [5], ecobiotechnology aims at applying “processes based on open mixed cultures and ecological selection principles (rather than genetic or metabolic engineering), thus combining the methodology of environmental biotechnology with the goals of industrial biotechnology.” Some recent studies have started to apply such principles also to the valorization of crude glycerol, showing interesting results, in terms of conversion efficiencies and decreased substrate and operating costs (no substrate pretreatment, no sterilization, etc.) mainly due to lower energy consumption [2, 4, 6, 7]. Glycerol fermentation can lead to the production of several useful metabolites, such as alcohols (i.e., ethanol and butanol), 1,3-propanediol (1,3 PD), 2,3-butanediol (2,3 BD), hydrogen, polyhydroxyalkanoates (PHA), and volatile fatty acids (VFAs) [8–13]. The latter represent important bulk chemicals and preferred substrates for many bioprocesses [14]. Interestingly, they are also known to be preferred substrates for enhanced polyhydroxyalkanoates (PHA) production [15] and in principle they might be used for a 2-stage process for the bioconversion of glycerol into VFAs, followed by PHA production.

Thus, in recent years, the glycerol glut problem has led to several studies on the conversion of crude glycerol. However, valorization of crude glycerol derived from second-generation (2G) biodiesel has been scarcely investigated and, to our knowledge, bioconversion of crude glycerol from the processing of animal fat derived biodiesel has been reported only by Sarma and colleagues [16] so far. On the other hand, production of 2G biodiesel is expected to increase in the near future, due to incentives. Europe, for instance, has proposed subsidies for the production of biofuels produced from waste feedstocks (i.e., “multiple accounting mechanism,” Renewable Energy Directive 2009/28/EC), thus leading to an enhanced production of crude glycerol derived from 2G biodiesel.

Nevertheless, the use of such a substrate, containing high amounts of contaminants such as soaps and long chain fatty acids (LCFA), salts, ashes, and methanol, can strongly interfere with, or even inhibit, the microbial growth and conversion efficiency, especially in the case of pure strains [17, 18]. In fact, crude glycerol derived from complex waste materials, such as meat processing and restaurant waste, is considered to have even more impurities (very high amount of sulfur and LCFA, very low pH, etc.) than the crude glycerol derived from pure substrates [16]. For this reason, most studies working with pure strains focus on the use of purified glycerol. This allows for higher substrate conversion efficiency but significantly increases processing costs [19]. A very important step to reduce costs related to the conversion of glycerol would therefore be to use crude glycerol directly, without previous pretreatment. This might be achieved by using selected mixed microbial cultures (MMCs). Since sterile cultivation enables an easy way of controlling microbial growth and product formation, most industrial biotechnological processes today are based on a single microbial strain. Nonetheless, there are many cases where the utilization of mixed cultures and/or cocultures appears to be advantageous over a single microorganism [20].

The ability of the selected MMC to create synergistic effects can help degrading complex substrates with different grades of impurities, also in nonsterile conditions. MMC can thus utilize a wide variety of complex substrates, rich in nutrients, but also potentially inhibiting effluents. This is particularly advantageous if industrial waste feedstock, containing compounds of undefined composition, are used [21]. In fact, unlike monocultures, MMCs show a complementary metabolism and are able to utilize different carbon sources. For this reason, they are considered by several authors to be of special interest in the fermentative processes [5, 22, 23], representing a promising alternative approach [5], in some cases even showing better performances than pure strains [24]. Therefore, a new promising direction in environmental biotechnology is to apply the principles of ecobiotechnology and adaptive laboratory evolution to develop a mixed microbial population, selected to achieve a higher production yield and which would have unique metabolic capacities [25], at lower operational costs [6, 26].

The objective of this study is the selection and adaptation of MMC, able to ferment crude glycerol generated from animal fat derived biodiesel and produce building-blocks and green chemicals. Various adaptation strategies have been investigated for the enrichment of suitable and stable MMCs, trying to overcome inhibition problems and enhance substrate degradation efficiency, as well as production of soluble fermentation products.

## 2. Material and Methods

### 2.1. Choice of Crude Glycerol

Unless differently stated, nonpretreated crude glycerol provided by Daka Biodiesel (Denmark), obtained from the transesterification of butchery waste (based on animal fat categories 1 and 2 according to the EU regulation number 1069/2009 and 142/2011), was used. The main characteristics of this type of crude glycerol are reported in Table 1.

### 2.2. Experimental Plan

The enrichment and selection were performed in small batches through repeated transfers of different inocula, in order to compare their performances. Each experiment was performed in triplicate. Activated sludge and anaerobic sludge were used as inoculum source. The latter underwent heat-shock treatment and the fermentation performance was compared to the nonpretreated sludge. Heat-shock allows selecting for spore forming bacteria (typically Gram-positive bacteria, such as Clostridia, which are abundant in anaerobic sludge and are well-known in dark fermentation processes), while getting rid of methanogens. Activated sludge instead is mainly made of enterobacteria, typically nonspore forming bacteria, which would be inhibited by the heat-shock. Enterobacteria are considered to be an important component in dark fermentation processes and the heat shock would lead to a reduction of additional fermentation pathways [27]. Moreover the activated sludge is not anaerobic and does not favor the growth of methanogens, and thus the heat-shock treatment would not be necessary or beneficial.

Two different growth media were used for the enrichment, containing 10 g/L glycerol: a medium rich in trace metals, vitamins, and growth factors (BA) and a Minimal Medium (MM), which does not include yeast extract, tryptone, vitamin, or mineral solutions. 

*Transfers*. 10% inoculum was used in 125 mL vials of 40 mL working volume. The experiments were performed to compare the efficiency of two enrichment strategies: (a) Kinetic Control (KC) and (b) non-Kinetic Control, in which the inoculum was transferred only at the End of Fermentation (EF).

**Figure 1:**
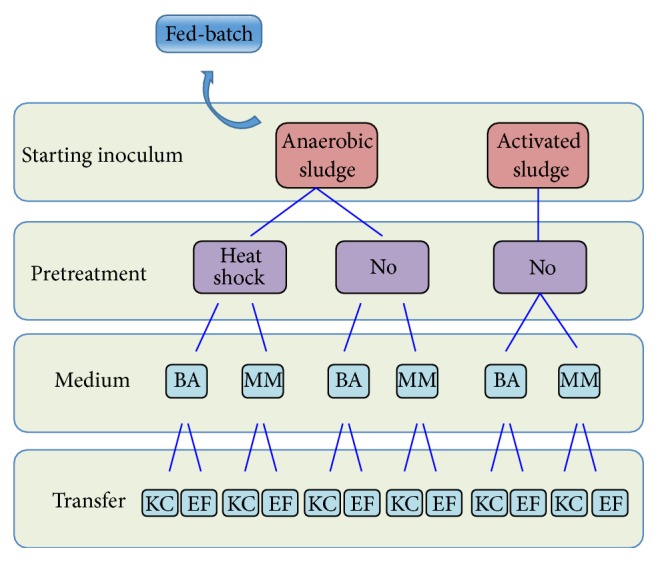
Transfer scheme for the selection and enrichment in batch conditions. KC = Kinetic Control; EF = End of Fermentation; MM = Minimal Medium; BA = BA medium.

*Kinetic Control*. Transfers occurred during the (late) exponential growth phase, in rapid successions (after 21 h fermentation).* End of Fermentation*. The transfers occurred after 72 h, when no more fermentation gases were produced. A scheme of the experimental inoculum transfers is presented in Figure 1. In addition, fed-batch experiments (400 mL working volume in 1 L serum bottle) and enrichment on hexane-pretreated crude glycerol were also performed, using anaerobic sludge as starting inoculum.

Liquid and gas samples were collected on a regular basis.

#### 2.2.1. Microorganisms Storage and Activation

MMCs obtained during the exponential growth phase were stored in the freezer at −18°C and periodically refreshed. Prior to use, the frozen mixed culture was transferred to the refrigerator at 4°C, for 2 hours and then for an additional hour at room temperature, before being inoculated. Activation was performed in the same conditions as the respective enrichment and 10% v/v inoculum was transferred into fresh medium after 21 hours (in case of Kinetic Control experiments) or 72 hours (in case of End of Fermentation experiments).

#### 2.2.2. Batch Experiments

125 mL serum vials were used for batch experimentation, to enrich the (activated or anaerobic) sludge through repeated transfers into fresh medium, according to the transfer scheme shown in Figure 1. 36 mL growth medium (either MM or BA medium), containing around 10 g/L glycerol, was flushed for 5 minutes with a mixture of 80% N_2_ and 20% CO_2_, in order to obtain anaerobic conditions, prior to inoculation, and incubated at 37°C, using an orbital shaker at 150 rpm. Gas and liquid samples were collected before transferring 10% v/v of fermentation broth (representing the new inoculum) into fresh medium. All transfer steps were performed in triplicate.

#### 2.2.3. Hexane Pretreatment of Crude Glycerol

Enrichment of (heat-shock treated) anaerobic sludge was also performed (in the same batch conditions described in Section 2.2.2) using hexane-pretreated crude glycerol. The extraction step was applied in order to reduce the concentration of lipids and (long chain) fatty acids present in the crude glycerol (coming from fat derived biodiesel) and evaluate its potential inhibitory effect on the microbial growth. Hexane pretreatment was performed as described by Anand and Saxena [28] and the batch transfers were performed with Kinetic Control (every 21 h).

#### 2.2.4. Fed-Batch Experiments

Repeated fed-batch culture was used for the enrichment of heat-shock treated anaerobic sludge, in a 1 L serum vial with 300 mL work solution, containing 90% anaerobic sludge and 10% BA medium, with around 10 g/L (nonpretreated) glycerol. The serum vial was flushed for 15 minutes with a mixture of 80% N_2_ and 20% CO_2_, in order to obtain anaerobic conditions, and incubated at 37°C and 150 rpm. Every day, an aliquot of around 30 mL was collected and substituted with an equivalent amount of fresh BA medium, containing 10 g/L glycerol. Gas and liquid samples were collected prior to this operation.

### 2.3. Media Composition

#### 2.3.1. Minimal Medium

Minimal Medium (MM) is a very simple growth medium, containing, per litre of distilled water: 10 g glycerol, 3.4 g K_2_HPO_4_·3H_2_O, 1.3 g KH_2_PO_4_, 2 g (NH_4_)_2_SO_4_, 0.2 g MgSO_4_·7H_2_O, 20 mg CaCl_2_·2H_2_O, and 5 mg FeSO_4_·7H_2_O [29].

For cultivation, 36 mL of medium was dispensed into 125 mL serum bottles and sealed with butyl rubber stoppers. Subsequently the medium was flushed with a mixture of nitrogen and CO_2_ (80 : 20 v/v) for 5 minutes and inoculated with 4 mL inoculum (10% v/v inoculum), before being incubated at 37°C with continuous stirring (150 rpm). Initial pH was 7.

#### 2.3.2. Rich Medium

A complete synthetic medium for anaerobes (referred to as BA medium [30]), which contains salts, vitamins, and trace elements beside pH buffers and reducing agents was also used. The medium was prepared from the following stock solutions (containing, per litre of distilled water): (A) 100 g NH_4_Cl, 10 g NaCl, 10 g MgCl_2_·6H_2_O, and 5 g CaCl_2_·2H_2_O; (B) 200 g K_2_HPO_4_·3H_2_O; (C) trace metal and selenite solution: 2 g FeCl_2_·4H_2_O, 0.05 g H_3_BO_3_, 0.05 g ZnCl_2_, 0.038 g CuCl_2_·2 H_2_O, 0.05 g MnCl_2_·4H_2_O, 0.05 g (NH_4_)_6_Mo_7_O_24_·4H_2_O, 0.05 g AlCl_3_, 0.05 g CoCl_2_·6H_2_O, 0.092 g NiCl_2_·6H_2_O, 0.5 g ethylenediaminetetraacetate, 1 mL concentrated HCl, and 0.1 g Na_2_SeO_3_·5H_2_O; (D) 52 g NaHCO_3_; and (E) vitamin mixture according to Wolin et al. [31].

974 mL of redistilled water was added to the following stock solutions: A, 10 mL; B, 2 mL; C, 1 mL; D, 50 mL; and E, 1 mL [30].

### 2.4. Inocula

Activated sludge was collected from the wastewater treatment plant of Daka Biodiesel, Denmark, as it was anticipated that it should be already enriched in microbes able to use glycerol and lipid substances as carbon source.

Anaerobic sludge was obtained from the Lundtofte Wastewater Treatment plant (Denmark) and supplemented with the effluent of a lab-scale anaerobic digester (50/50 v/v), treating swine manure.

The heat-shock pretreatment was obtained by heating the anaerobic sludge mixture for 15 minutes at 90°C, while flushing with the N_2_-CO_2_ mixture.

### 2.5. Analytical Methods

Detection and quantification of glycerol, ethanol, 1,3-propanediol, and lactic acid were obtained with a HPLC equipped with a refractive index and Aminex HPX-87H column (BioRad) at 60°C. A solution of 4 mM H_2_SO_4_ was used as an eluent at a flow rate of 0.6 mL/min.

Samples for HPLC analysis were centrifuged at 10,000 rpm for 10 min, filtered through a 0.45 *μ*m membrane filter, and finally acidified with a 10% w/w solution of H_2_SO_4_.

For the quantification of volatile fatty acids (VFAs), filtered samples were acidified with H_3_PO_4_ (30 *μ*L of 17% H_3_PO_4_ was added in 1 mL of sample) and analyzed on a gas chromatograph (PerkinElmer, Clarus 400), equipped with a flame ionization detector and a capillary column (Agilent HP-FFAP, 30 m long, 0.53 mm inner diameter). The oven was programmed to start with 105°C (for 3 minutes), followed by a ramp that reaches 130°C at a rate of 8°C/min and subsequently 230°C (held for 3 min) at a rate of 45°C/min. Nitrogen was used as the carrier gas at 13 mL/min; the injector temperature was set at 240°C and the detector at 230°C.

The total volume of gas production was measured using a water displacement system [32].

Hydrogen content in the produced gas was measured with a gas chromatograph (SRI GC model 310) equipped with a thermal conductivity detector and a packed column (Porapak-Q, length 6 ft and inner diameter 2.1 mm). The volume of H_2_ produced in sealed vials during glycerol fermentation tests was calculated by the mass balance equation [33].

Multivariate data analysis was performed using Unscrambler X 10.1 software (by Camo). A Principal Component Analysis (PCA) [34] was chosen as a tool to explore the big data matrix obtained from the main fermentation parameters monitored during the enrichments.

### 2.6. Next Generation Sequencing

DNA was extracted from the pellets of 5 mL crude samples using the PowerSoil DNA Isolation Kit (MoBio) according to the standard procedure. Sequencing amplicon libraries were generated by PCR following the “16S Metagenomic Sequencing Library Preparation, Preparing 16S Ribosomal RNA Gene Amplicons for the Illumina MiSeq System” protocol (Illumina part number 15044223 rev. B). Internal parts of the 16S ribosomal RNA (rRNA) gene, covering variable regions V3 and V4, were PCR-amplified with the KAPA HiFi HotStart ReadyMix (KAPA Biosystems) and the primers 5′-TCGTCGGCAGCGTCAGATGTGTATAAGAGACAGCCTACGGGNGGCWGCAG-3′ and 5′-GTCTCGTGGGCTCGGAGATGTGTATAAGAGACAGGACTACHVGGGTATCTAATCC-3′ and purified with the Agencourt AMPure XP kit (Beckman Coulter Genomics). The Nextera XT Index Kit was used to add sequencing adapters and multiplexing indices. Pooled DNA libraries were sequenced on a MiSeq sequencer (Illumina) using the MiSeq Reagent Kit v3 in the 2·300 bp paired-end mode.

Sequencing reads were demultiplexed, trimmed, and OTU-classified using the Metagenomics Workflow of the MiSeq Reporter Software v.2.3 (Illumina). This workflow uses an Illumina proprietary classification algorithm and an Illumina-curated version of the Greengenes 13.5 (May 2013) taxonomy database, which covers 3 kingdoms, 33 phyla, 74 classes, 148 orders, 321 families, 1086 genera, and 6466 species.

Due to the relatively high number of unclassified reads found at the species level, comparisons between samples are presented at the genus level, while comparisons at the species, family, order, class, and phylum level are available as supplementary information (in Supplementary Material available online at http://dx.doi.org/10.1155/2015/932934). Sequencing reads have been deposited to the sequence read archive of NCBI under the Bioprojects PRJNA285034 (http://www.ncbi.nlm.nih.gov/bioproject/285034) and PRJNA284929 (http://www.ncbi.nlm.nih.gov/bioproject/284929).

## 3. Results and Discussion

### 3.1. Enrichment in Batch Conditions

#### 3.1.1. Activated Sludge

Based on the experimental scheme (Figure 1), 12 different selection conditions were tested in triplicate. The enrichment using activated sludge showed good results in terms of substrate degradation, and it continued unhindered for several transfers, with no evident inhibition (due to the use of crude glycerol). This actually indicated the possibility to increase the substrate concentration in future studies. The best results obtained, in terms of substrate degradation efficiency (practically reaching 100%) and biogas production, were observed with MM-KC. This experimental condition led to the highest ethanol production, converting about 10 g/L glycerol in 21 h (maximum yield = 4.6 g/g), with a concomitant 1,3 PD yield of approximately 3 g/g. After 16 transfers, however, the distribution of the main metabolites changed, with 1,3 PD becoming the dominant one, and showing an increase in butyrate during the last transfers.

MM-EF also showed a high substrate degradation efficiency and (with exception of transfers 5–7) the main metabolites were represented by 1,3 PD and butyrate. This condition performed the best butyrate production, with a maximum yield of 3.3 g/g (from 8.5 g/L glycerol in 72 h fermentation), together with 1,3 PD yield of 4.7 g/g.

The use of BA medium (experiments 3 and 4) seemed not to favor solventogenesis pathway (almost no ethanol production was observed), while 1,3 PD was still by far the main metabolite (with an average production of 3.67 ± 0.56 g/L and 3.99 ± 0.74 g/L for KC and EF, resp.), followed by butyrate and acetate. Also in this case, the End of Fermentation seemed to favor butyrate production, with a yield reaching up to 2.99 g/g (from 7.7 g/L glycerol in 72 h fermentation) in BA-EF.

Hydrogen % in the biogas was rather modest in all experiments, reaching in most cases around 20%.

**Figure 2:**
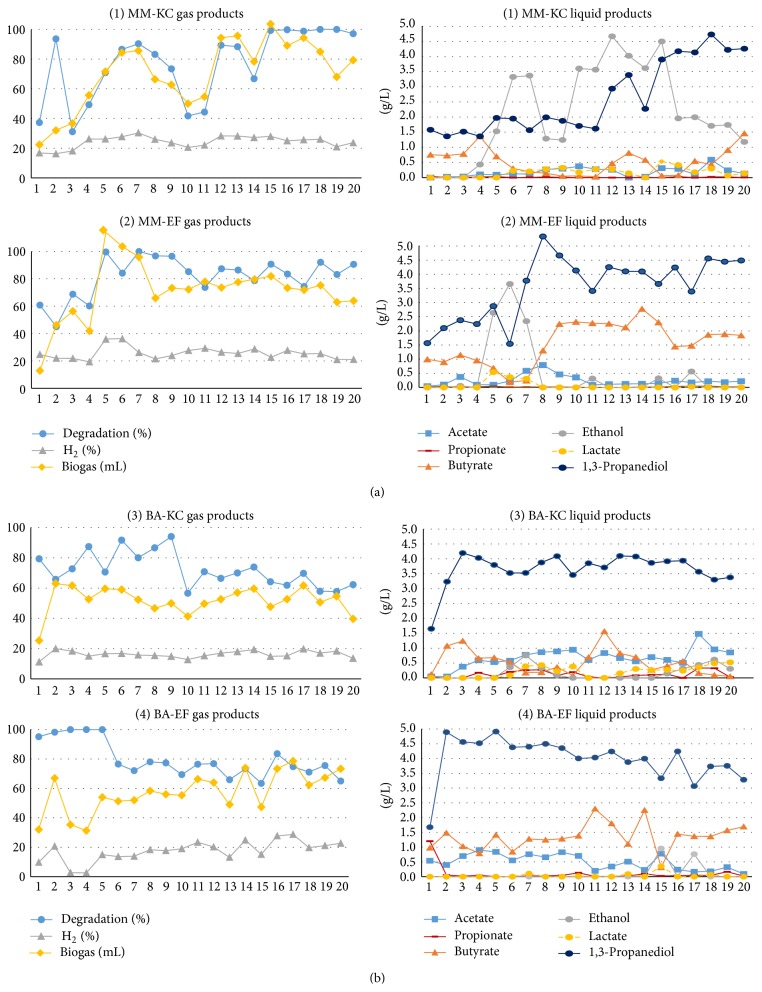
Fermentation products monitored during the enrichment of activated sludge in batch conditions, through repeated transfers using MM (a) and BA (b) medium. (1) MM-KC = Minimal Medium with Kinetic Control (21 h); (2) MM-EF = Minimal Medium with End of Fermentation (72 h); (3) BA-KC = Basal Medium with Kinetic Control (21 h); (4) BA-EF = Basal Medium with End of Fermentation (72 h).

The distribution of main metabolites and substrate degradation (%) observed during the enrichment process with activated sludge are shown in Figures 2(a) and 2(b).

**Figure 3:**
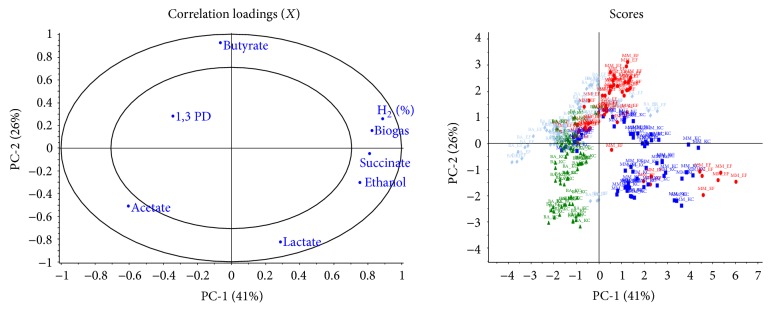
Principal Component Analysis showing the distribution of the main fermentation parameters (correlation loading plot) and the distribution of the samples (score plot) during the experiments with activated sludge: MM-EF (in red), MM-KC (in blue), BA-EF (in grey), and BA-KC (in green). The first two components explain together about 67% of the total variability.

Principal Component Analysis, based on the complete data matrix of 240 samples with 11 variables, showed clear differences between the tested enrichment strategies (Figure 3), with EF closer related to butyrate (especially MM-EF) and BA-KC closer related to acetate. In general, the first Principal Component (PC) showed an increase of ethanol and hydrogen, moving towards the right, while the second PC showed an increase of butyrate production moving upwards. The first PC roughly separated EF and KC (with the exception of MM-EF), while the second PC separated MM from BA.

**Figure 4:**
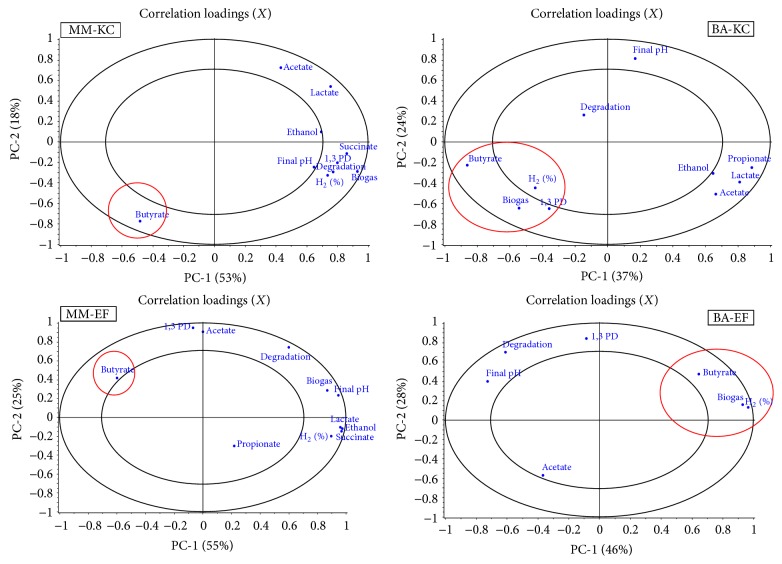
Principal Component Analysis showing the distribution of the main fermentation parameters (correlation loading plot) of the four experimental conditions (namely, MM-EF, MM-KC, BA-EF, and BA-KC) separately, during the experiments with activated sludge. The first two components explain together more than 60% of the total variability, in all cases.

Furthermore, a comparison of the correlation loadings obtained with the data of the four enrichment conditions (MM-KC, MM-EF, BA-KC, and BA-EF) separately, showed that, only in the case of BA, butyric acid was related to H_2_ production (Figure 4), as would be expected from a direct glycerol conversion into butyrate. In fact, glycerol conversion to butyric acid has a theoretically yield of 2 mol/mol [35].

Interestingly, in the case of MM, butyrate production was negatively correlated with lactic and acetic acid, and also with hydrogen in MM-EF, while it was positively correlated with hydrogen production when using BA medium, thus implying a secondary fermentation (*sensu* Agler et al. [21]) (a butyrate production which does not come directly from glycerol conversion).

There might be several possible pathways, leading to butyrate production through the conversion of lactate and acetate [36], besides the above-mentioned conversion of glycerol. Some examples are provided in

(1) $$\begin{array}{c} \text{Lactate}+0.4\;\text{Acetate}+0.7H^{+}⟶0.7\;\text{Butyrate}+0.6H_{2}+CO_{2}+0.4H_{2}O\;\Delta G=-183.9 \end{array}$$

(2) $$\begin{array}{c} \text{Lactate}+\text{Acetate}+H^{+}⟶\text{Butyrate}+0.8H_{2}+1.4CO_{2}+0.6H_{2}O\;\Delta G=-59.4 \end{array}$$

(3) $$\begin{array}{c} 2\text{Lactate}+H^{+}⟶\text{Butyrate}+2H_{2}+2CO_{2}\;\Delta G=-64.1 \end{array}$$

It is also worth noting that Zhu and Yang [37] observed a metabolic shift from butyrate formation to lactate and acetate at pH < 6.3, associated with decreased activities of phosphotransbutyrylase and NAD-independent lactate dehydrogenase, and increased activities of phosphotransacetylase and lactate dehydrogenase. Our batch experiments were operated without pH control, starting at pH 7 and typically ending at around 4.8, due to glycerol acidification. Therefore it is likely that such a metabolic shift was also involved in our fermentation tests.

#### 3.1.2. Anaerobic Sludge

Differently from activated sludge, the enrichment of anaerobic sludge in batch conditions showed a clear inhibition, regardless of the selection strategy (BA and MM growth medium, EF or KC transfers). The inhibition was presumably related to the high concentration of LCFA and the negative interaction with the cell membranes of Gram-positive anaerobic bacteria of the anaerobic sludge, rather than product inhibition. In fact, even after centrifuging the inoculum, washing away the supernatant and resuspending the pellet into fresh medium (thus washing away extracellular soluble metabolites), no recovery of the fermentation was achieved. Addition of specific elements such as yeast extract or vitamin and mineral solution did not have any effect either.

**Figure 5:**
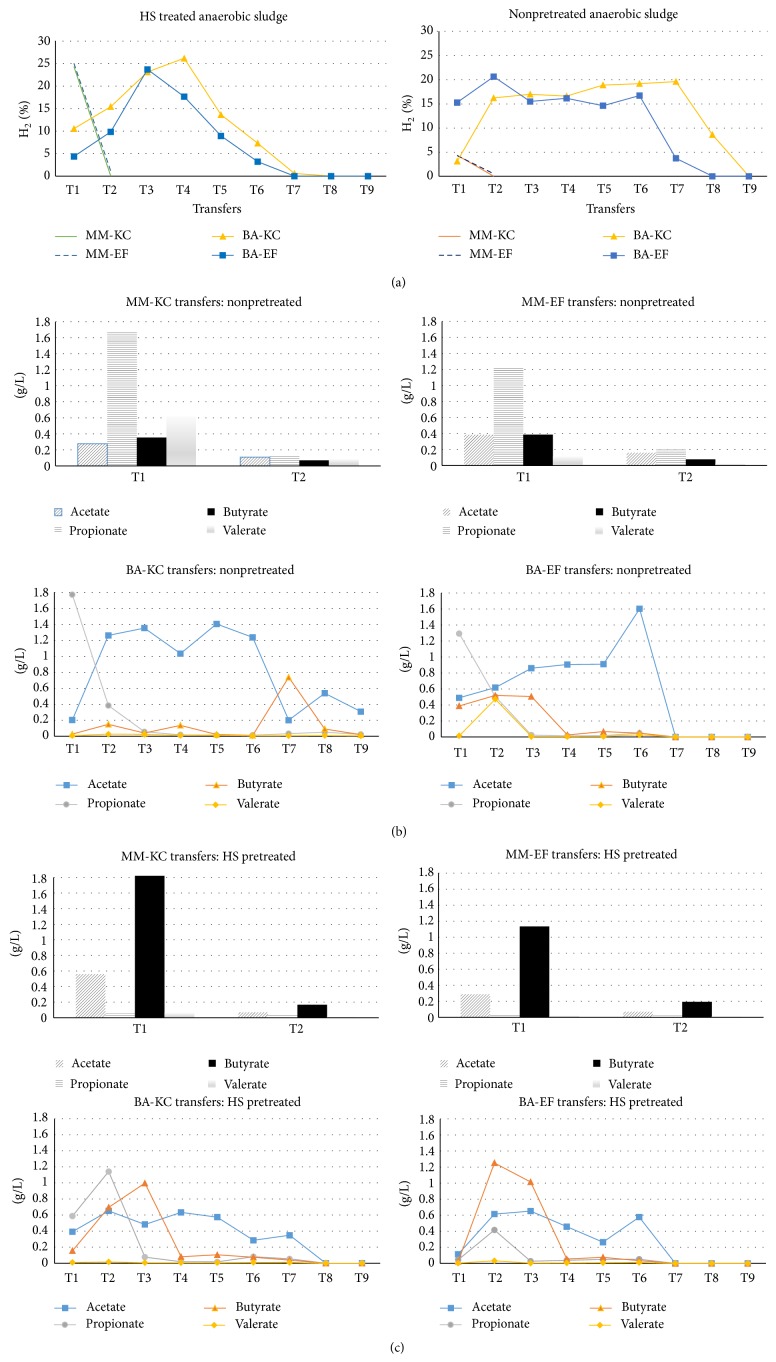
Results of batch transfers, during the enrichment of anaerobic sludge, showing H2% (a) in the headspace, soluble metabolites from nonpretreated anaerobic sludge (b), and soluble metabolites from heat-shock treated anaerobic sludge (c). MM-KC = Minimal Medium with Kinetic Control (21 h); MM-EF = Minimal Medium with End of Fermentation (72 h); BA-KC = Basal Medium with Kinetic Control (21 h); BA-EF = Basal Medium with End of Fermentation (72 h).

The distribution of main metabolites and fraction of H_2_ (in the headspace) detected during the enrichment process with anaerobic sludge are shown in Figure 5. The use of MM (without nutrient supplements) led to inactivation after only 1 transfer, while BA reached 6-7 transfers before being inhibited (Figure 5(a)). Nonpretreated sludge (Figure 5(b)) showed a high production of propionic acid, while, with heat-shock treated sludge (Figure 5(c)), butyric acid was the dominant metabolite. The latter condition was chosen for an alternative selection strategy, using fed-batch conditions.

#### 3.1.3. Hexane-Pretreated Glycerol Tests

As mentioned above, heat-shock treated (HS) inoculum was chosen for further experimentation. The possible inhibiting effect of LCFA and “lipidic compounds” was evaluated in the following test. The hypothesis was that the animal fat derived crude glycerol would contain inhibiting amounts of LCFA, which might negatively interfere with the membrane of Gram-positive bacteria of the anaerobic sludge. Activated sludge was not included in this test, since it did not show any inhibition.

Nonextracted crude glycerol showed an organic carbon content, expressed as chemical oxygen demand (COD), of 1309 ± 32 g COD/L, while the extracted crude glycerol was 1172 ± 12 g COD/L, thus suggesting that approximately 137 g COD/L of “lipidic compounds” was removed (which would approximately correspond to 34.7 g/L of oleic acid, a typical LCFA known for its inhibiting effect).

**Figure 6:**
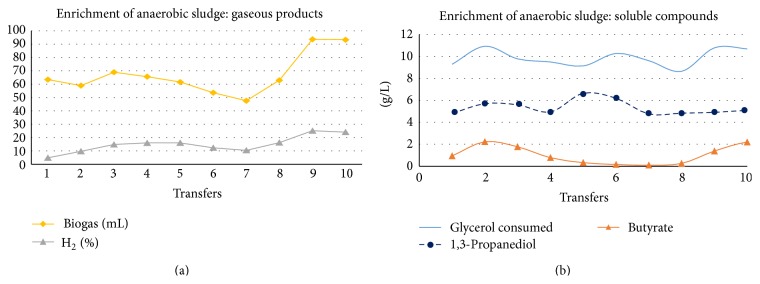
Results from the batch transfers of anaerobic sludge, using hexane-treated crude glycerol, showing gas products (a) and glycerol consumption, together with the main soluble metabolites (b).

As can be seen in Figure 6, repeated transfers in batch conditions with the hexane-treated crude glycerol led to high substrate degradation efficiency and the MMC was never inactivated, showing glycerol fermentation performances comparable with those obtained with activated sludge. This implied that, indeed, the inactivation of anaerobic sludge depended on the high LCFA content of the 2G crude glycerol.

However, since the aim of this study was the selection of MMC that can grow on nonpretreated crude glycerol, the possibility to achieve enrichment and adaptation tests of anaerobic sludge using fed-batch conditions was investigated.

### 3.2. Enrichment in Fed-Batch Conditions

**Figure 7:**
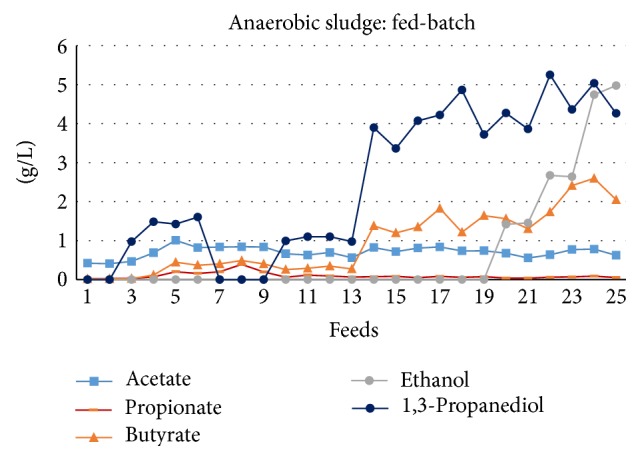
Distribution of main soluble metabolites observed during the fed-batch enrichment process with heat-shock treated anaerobic sludge.

As can be seen in Figure 7, the fed-batch operations allowed effective overcoming of crude glycerol inhibition with anaerobic sludge, leading to a good substrate conversion into mainly 1,3 PD, ethanol, and butyrate (after about 14 feedings). However, the reactor started to develop a community of sulfate reducing bacteria (SRB) that inhibited fermentation after roughly 7 feedings. For this reason, the sludge underwent a second heat-shock treatment (at 10 feedings) to allow further glycerol fermentation. Nonetheless, H_2_S production occurred again after 21 feedings. Probably, continuous mode fermentation with short hydraulic retention time (HRT) would thus represent a suitable approach for successful adaptation/enrichment of anaerobic sludge to untreated crude glycerol (possibly helping to rinse out slower growing SRB). For this reason, ongoing work is now focusing on identification of the operating parameters for maintaining a stable MMC in continuous mode and statistical optimization of key parameters for green chemicals production. Since activated sludge was successfully enriched in batch conditions, there was no need to perform fed-batch tests with this inoculum.

### 3.3. Molecular Characterization of the MMC during the Enrichment Process

The development of the MMC was monitored by sequencing amplicons of the V3 and V4 variable regions of the 16S rRNA gene. Operational taxonomic units (OTUs) were then assigned from each sequencing read and used as a measure of the microbial diversity of each sample. The copy number of the 16S rRNA gene varies from 1 to 15, depending on the species, and the OTUs are therefore only providing an estimate of the true microbial diversity. The copy number is varying but is relatively high in the taxa Firmicutes and Gammaproteobacteria, with a mean of 5.8 ± 2.8 copies, while it is lower for Bacteroidetes (3.5 ± 1.5), Betaproteobacteria (3.3 ± 1.6), Actinobacteria (3.1 ± 1.7), and Spirochaetes (2.4 ± 1.0) [38]. Overall, the Firmicutes and Gammaproteobacteria are overestimated in the analysis and the cell-count may for some genera be ~5–10-fold lower than the OTU count.

#### 3.3.1. Activated Sludge Experiments

In all these samples there was a dominance of bacteria belonging to the phylum Firmicutes, in particular from the classes Clostridia and Bacilli and of the class Gammaproteobacteria (Figures S1–S6). 

*MM-KC*. The enrichment was characterized by a strong decrease of the genera* Clostridium* and* Lactobacillus*, both Firmicutes, and an increase of* Klebsiella *and* Escherichia*, both Gammaproteobacteria (Table 2; Figure S2). In particular, the joint increase of the latter two probably favored an enhanced ethanol production (T10 and T13), while the dominance of* Klebsiella* alone (T18) was associated with a metabolic shift towards 1,3 PD (see Figure 2(a)). These results are in good agreement with previous observations with enriched activated sludge, selected with Kinetic Control [39]. 

*MM-EF*. The distribution of the main genera observed during these tests showed a sequence of dominance shifts, going from* Escherichia *to* Klebsiella*, and finally to* Clostridium *and* Escherichia*. The ethanol peak observed in T6 is associated with the dominance of* Escherichia* (around 55%), while the subsequent increase of* Klebsiella* (reaching almost 70%) shifted towards 1,3 PD production (T8: 5.2 g/L 1,3 PD and no ethanol production). Moreover, the stability of the community from T8 to T15 is also reflected in the distribution of the main metabolites (see Figure 2(a)). The higher butyric acid production observed after T7 might be related to the increase of the genus* Clostridium*, which includes several butyric acid producing species. 

*BA-KC*. Interestingly, a clear increase in biodiversity could be observed during the enrichment of BA-KC, with an initial dominance of* Clostridium* (86%) and a sharp decrease over time, leading to less than 8%. This decrease is associated with a concomitant increase of other genera, such as* Escherichia* (reaching 34%),* Lactobacillus *(13%), and a number of unclassified genera (approximately 14% in total, primarily from the classes Gammaproteobacteria and Clostridia; Figure S5), followed by* Serratia* and* Klebsiella* (10%). Higher butyric acid was observed in T1 and T12 in the presence of at least 70% of* Clostridium*, while an increased acetic acid production was observed in T18. 

*BA-EF*. In general, this enrichment was characterized by a dominance of* Clostridium, *with a decrease towards the last transfers. A decrease of acetic acid, and concomitant increase in butyric acid, could be observed comparing the samples T7 and T11, which were associated with a decrease of the genus* Slackia *(typically producing acetic acid and lactic and formic acid [40]) and an increase in* Clostridium*. A very sharp decrease of butyric acid (together with an increase in acetic acid and ethanol) could be observed in T15, which was associated with a decrease in* Clostridium* and a concomitant increase of unclassified genera, primarily belonging to the phylum Proteobacteria and in particular the class Gammaproteobacteria (Figures S5 and S6).

#### 3.3.2. Anaerobic Sludge Experiments

This subparagraph reports the results of MMC taxonomical characterization for the anaerobic sludge enriched on hexane-pretreated crude glycerol in batch tests (HT) and with the untreated crude glycerol in fed-batch (Figures S7–S12). Anaerobic sludge grown on untreated glycerol underwent quick inhibition and was thus not analyzed.

The main difference that can be observed between the batch and fed-batch conditions was the dominant presence of* Blautia* (up to 50%) in the latter (Table 3). The fed-batch community was also characterized by the genus* Clostridium,* in addition to a number of unclassified genera, primarily of the phylum Firmicutes. Dominant genera in batch conditions (HT) at T0 were* Clostridium* and unclassified genera (both around 30%), with an increase of* Clostridium *(reaching more than 45%) and* Klebsiella* (almost 30%) in T9. It is worth noting that T0 was a highly diverse sample, with multiple genera having abundances in the range of 0.1–0.9%, explaining why the total fraction only reached about 75% (see Figure S8). The unclassified genera found in T0 mainly belonged to the phyla Proteobacteria (in particular to the class Deltaproteobacteria) and Firmicutes (especially to the class Clostridia) (Figures S11 and S12).

A total of 19 genera belonging to SRB were retrieved in the different anaerobic sludge samples, even though always at a very low % (far below the cut-off set at 1%). Initial sludge (HS_T0) contained 18 different genera (mainly* Desulfovibrio *and* Desulfofrigus*), accounting for 1.19%, which decreased to 10 genera (0.0023%) in T9. This suggests that the Kinetic Control was effective in enriching faster growing (glycerol consuming) bacteria, such as* Clostridium* and* Klebsiella *species over SRB. In fed-batch conditions, instead, the absence of a Kinetic Control allowed the growth of SRB. Thus, even though a second heat-shock treatment (T11) was able to decrease SRB from initial 19 genera to 16 (accounting for 0.59%), this was probably sufficient to allow SRB to grow in the following weeks of fed-batch experimentation, as witnessed by the H_2_S production observed in the fed-batch reactor (which turned black and was characterized by the typical strong H_2_S smell). The most abundant genus found in T11 was* Desulfotomaculum* (mainly with the species* D. halophilum*).* Desulfotomaculum* comprises endospore forming, Gram-positive bacteria.* Desulfotomaculum* spp. are able to grow autotrophically (using H_2_/CO_2_) and produce sulfide and acetate. Besides H_2_ as electron donor, they are able to utilize alcohols and organic acids, which were likely to accumulate in the fed-batch system. Besides sulfate reduction they may also use various other sulfur compounds [41].

## 4. Conclusions

The selection and adaptation of* activated sludge* inoculum through successive transfers in batch conditions were performed successfully and continued unhindered for several months. The best results showed a substrate degradation efficiency of almost 100% (about 10 g/L) and different dominant metabolic products were obtained, depending on the selection strategy (mainly 1,3 PD, ethanol, or butyrate). In particular, the strategy of Kinetic Control coupled with Minimal Medium (MM-KC) led to a maximum ethanol yield of 4.6 g/L, together with a 1,3 PD yield of around 3 g/g, with complete substrate degradation within 21 h. The End of Fermentation coupled with Minimal Medium (MM-EF) showed a degradation efficiency of around 90–95%, with a maximum butyric acid yield of 3.3 g/g (from 8.5 g/L glycerol in 72 h fermentation), together with a 1,3 PD yield of 4.7 g/g. Tests with the rich BA medium showed a general lower substrate degradation efficiency but were also characterized by a high 1,3 PD and butyric acid production. Multivariate data analysis showed clear differences between different strategies and further suggested that only in the case of BA medium the butyric acid was directly produced from glycerol. In addition, End of Fermentation enrichment seemed to favor butyric acid production. On the other hand,* anaerobic sludge* (both, heat pretreated and not) exhibited inactivation after a few transfers in batch conditions, probably due to the presence of high concentration of lipidic compounds. Fed-batch mode turned out to be a valid alternative adaptation strategy, overcoming inhibition problems related to crude glycerol composition but was also associated with H_2_S production, thus implying the use of continuous mode to better select and adapt anaerobic sludge to the conversion of animal fat derived crude glycerol. After overcoming inhibition problems, main metabolites produced were comparable with those obtained with activated sludge, with a high 1,3 PD and butyric acid production.

Next Generation Sequencing represented a useful tool to monitor the changes in microbial composition of MMCs, highlighting the development of a glycerol consuming community (with numerous strains belonging to the genera* Clostridium*,* Klebsiella,* and* Escherichia*), thus confirming the effectiveness of the enrichment strategy.

## Supplementary Material

Graphical representation of the change in composition of mixed microbial cultures, at the major levels of taxonomic classification (species, genus, family, order, class, and phylum). Results from batch transfer during the enrichment of activated sludge, and for the anaerobic sludge enriched on hexane-pretreated crude glycerol in batch tests and with the untreated crude glycerol in fed-batch are shown separately. Data from the genus level is also presented in tables 2 and 3.

## Figures and Tables

**Table 1:** Crude glycerol characteristics.

Content	Typical values
Raw glycerine	75%
Fat	10%
Methanol	<1%
Sulphur	1-2%
Moisture	10%
Ash	5%
Density	1.2–1.3 Kg/L
pH	1.5

**Table 2:** Metagenomic classification of the MMC at the genus level. Results of batch transfers, during the enrichment of activated sludge, expressed as fraction (%). MM-KC = Minimal Medium with Kinetic Control (21 h); MM-EF = Minimal Medium with End of Fermentation (72 h); BA-KC = Basal Medium with Kinetic Control (21 h); BA-EF = Basal Medium with End of Fermentation (72 h). T0–T20 = transfer numbers. ND = Not detected. Genera appearing at frequencies below 1% in all samples were omitted.

GENERA	MM-KC							MM-EF							BA-KC				BA-EF				
	T1	T3	T7	T10	T13	T18		T0	T6	T7	T8	T15	T20		T1	T12	T18		T0	T7	T11	T15	T20
	%	%	%	%	%	%		%	%	%	%	%	%		%	%	%		%	%	%	%	%
*Clostridium*	51.3	80.8	37.0	12.4	28.8	18.1		28.4	1.42	1.21	40.1	43.2	83.9		86.4	67.4	7.92		67.9	60.3	73.7	32.0	45.4
*Klebsiella*	0.74	0.42	47.4	28.9	19.1	65.4		28.0	0.13	66.7	0.15	0.07	0.03		0.30	9.12	9.18		0.02	0.03	0.19	6.61	0.03
*Escherichia*	0.54	8.46	0.60	33.5	28.7	1.05		0.99	54.2	7.05	31.0	31.6	0.05		0.74	5.16	34.4		0.05	0.11	0.84	30.9	42.8
*Unclassified*	6.47	2.83	8.31	8.14	11.5	8.88		13.8	18.4	10.5	13.5	10.9	1.23		2.91	6.56	14.2		18.3	3.41	5.93	5.72	4.03
*Lactobacillus*	29.7	0.07	0.11	0.01	0.02	0.01		0.39	3.53	0.78	0.79	4.74	14.3		6.05	4.72	13.3		0.01	16.8	14.8	21.8	4.12
*Slackia*	0.07	<0.01	<0.01	<0.01	0.01	<0.01		0.01	7.04	1.21	5.16	0.41	0.03		<0.01	3.61	0.55		1.05	18.1	2.61	0.21	1.01
*Serratia*	0.01	2.06	0.45	9.97	5.72	0.59		0.64	8.02	2.68	4.36	4.35	0.01		0.28	1.05	10.3		0.01	0.01	0.14	1.73	1.35
*Enterobacter*	0.01	1.58	0.93	3.86	3.72	1.39		0.66	3.67	1.78	2.27	2.50	<0.01		0.08	0.55	2.61		<0.01	<0.01	0.07	0.74	0.25
*Alkaliphilus*	0.29	0.01	0.02	<0.01	<0.01	0.01		3.66	0.01	<0.01	0.01	0.01	<0.01		0.14	<0.01	0.01		0.65	<0.01	0.02	ND	ND
*Tolumonas*	0.01	0.22	1.74	1.19	0.70	2.73		1.43	0.04	2.66	0.03	0.03	ND		0.06	0.48	0.63		<0.01	<0.01	0.01	0.01	<0.01
*Negativicoccus*	0.02	<0.01	0.01	<0.01	<0.01	<0.01		0.01	<0.01	0.00	<0.01	<0.01	<0.01		0.01	0.14	2.56		0.06	0.02	0.04	<0.01	ND
*Blautia*	0.20	0.05	0.57	0.04	0.01	0.04		0.92	0.07	0.02	0.01	0.01	<0.01		0.10	0.01	0.29		0.03	0.01	0.01	ND	ND
*Ruminococcus*	ND	ND	ND	<0.01	ND	ND		0.02	0.13	2.17	0.14	0.13	ND		<0.01	<0.01	<0.01		0.05	0.24	ND	ND	ND
*Erwinia*	<0.01	2.11	0.06	0.10	0.06	0.07		0.03	0.05	0.46	0.03	0.04	ND		<0.01	0.04	0.25		ND	<0.01	<0.01	0.05	<0.01
*Methylotenera*	0.91	0.05	<0.01	<0.01	ND	<0.01		0.89	ND	<0.01	ND	ND	<0.01		0.10	ND	<0.01		1.97	ND	ND	ND	ND
*Geobacillus*	0.14	0.02	0.05	0.03	<0.01	0.05		1.44	<0.01	0.02	<0.01	<0.01	<0.01		0.05	<0.01	0.03		0.03	<0.01	<0.01	ND	ND
*Pseudomonas*	0.73	0.12	0.06	0.18	0.04	0.01		1.16	0.04	0.02	0.04	0.03	0.01		0.02	0.01	0.01		0.01	<0.01	0.02	0.01	0.27
*Weissella*	1.08	0.02	<0.01	<0.01	<0.01	<0.01		0.55	<0.01	<0.01	<0.01	<0.01	ND		0.21	<0.01	0.01		0.41	<0.01	0.01	ND	ND

**Table 3:** Metagenomic classification of the MMC at the genus level, for the anaerobic sludge enriched on hexane-pretreated crude glycerol in batch tests (HT) and with the untreated crude glycerol in fed-batch, expressed as fraction (%). T0–T11 = transfer numbers. ND = Not detected. Genera appearing at frequencies below 1% in all samples were omitted.

Genera	HT		FED-BATCH
	T0	T9	T11
	%	%	%
*Blautia*	0.24	0.04	50.8
*Clostridium*	30.1	46.6	16.2
*Unclassified*	31.5	6.45	9.89
*Klebsiella*	0.01	28.8	0.02
*Escherichia*	0.06	10.3	<0.01
*Enterococcus*	0.02	0.27	6.19
*Alkaliphilus*	5.64	0.06	0.88
*Soehngenia*	<0.01	ND	3.52
*Serratia*	0.01	2.67	0.04
*Pedobacter*	2.38	0.02	0.08
*Enterobacter*	0.02	2.21	0.01
*Propionispora*	1.99	0.01	0.03
*Treponema*	1.42	0.01	0.03
*Peptoniphilus*	0.07	0.02	1.35
*Flavobacterium*	1.33	0.03	0.54
*Sedimentibacter*	0.33	<0.01	1.26
