# Corrigendum to “Comparison of Different Strategies for Selection/Adaptation of Mixed Microbial Cultures Able to Ferment Crude Glycerol Derived from Second-Generation Biodiesel”

**DOI:** 10.1155/2017/7602495

**Published:** 2017-02-06

**Authors:** C. Varrone, T. M. B. Heggeset, S. B. Le, T. Haugen, S. Markussen, I. V. Skiadas, H. N. Gavala

**Affiliations:** ^1^Department of Chemistry and Biosciences, Aalborg University, 2350 Copenhagen, Denmark; ^2^Department of Chemical and Biochemical Engineering, Technical University of Denmark, 2800 Lyngby, Denmark; ^3^Biotechnology and Nanomedicine, SINTEF Materials and Chemistry, 7465 Trondheim, Norway

In the article titled “Comparison of Different Strategies for Selection/Adaptation of Mixed Microbial Cultures Able to Ferment Crude Glycerol Derived from Second-Generation Biodiesel” [1], there is a systematic error in the yields reported (in g/g) in section “3.1.1. Activated Sludge.” It is reported again in section “4. Conclusions.” These yields need to be divided by a factor of 10, due to a conversion mistake. This does not alter or affect the discussions and conclusions that have been reached in this paper, since the scope was to make a comparison among the different mixed cultures obtained.
