# Supplementary material for: Comparison of Different Strategies for Selection/Adaptation of Mixed Microbial Cultures Able to Ferment Crude Glycerol Derived from Second-Generation Biodiesel
Source: Biomed Res Int. 2015 Oct 5;2015:932934. doi: 10.1155/2015/932934 (PMC4609794; doi:10.1155/2015/932934)
Supplement: Supplementary file 1 — Graphical representation of the change in composition of mixed microbial cultures, at the major levels of taxonomic classification (species, genus, family, order, class, and phylum). Results from batch transfer during the enrichment of activated sludge, and for the anaerobic sludge enriched on hexane-pretreated crude glycerol in batch tests and with the untreated crude glycerol in fed-batch are shown separately. Data from the genus level is also presented in tables 2 and 3. [file 932934.f1.pdf]

SUPPLEMENTARY MATERIAL

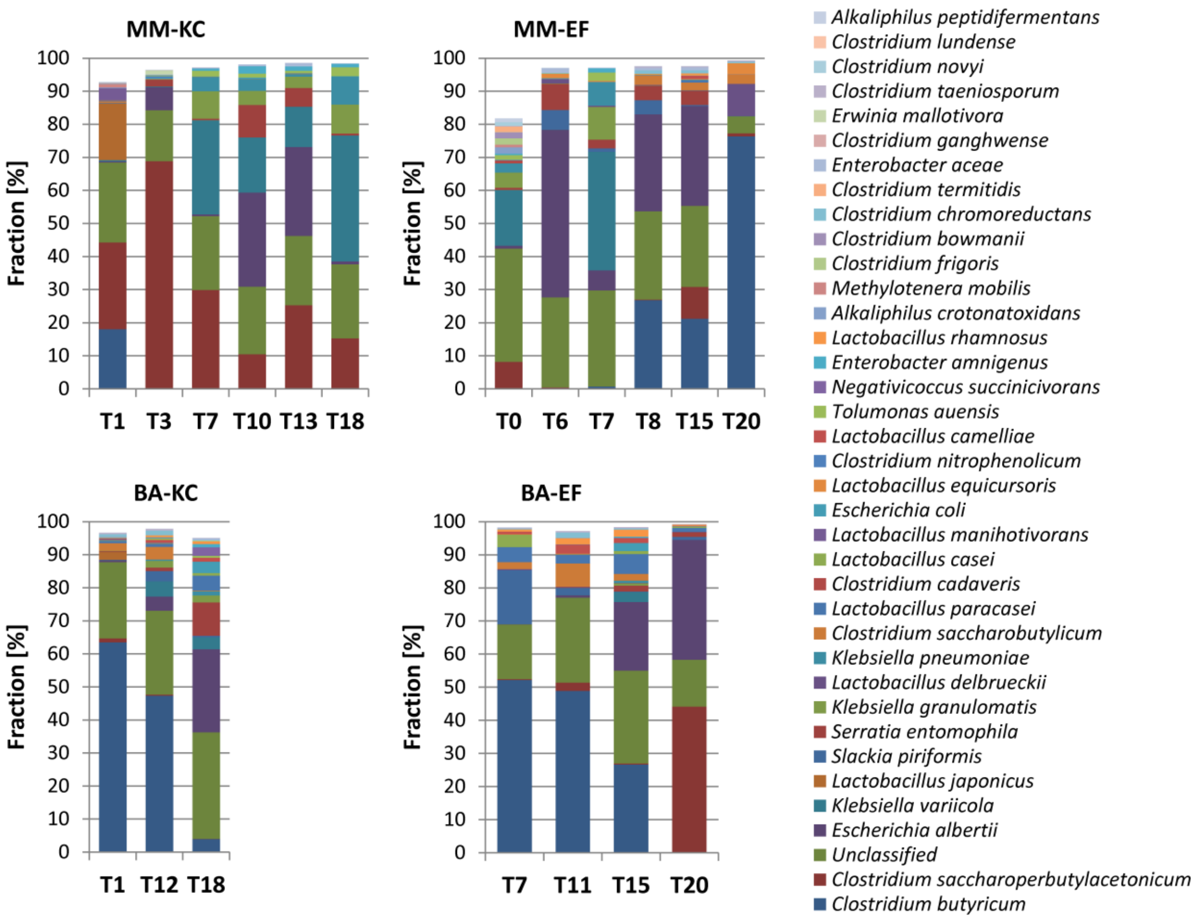

S1. Metagenomic classification of the MMC at the species level. Results of batch transfers, during the enrichment of activated sludge. MM-KC = Minimum Medium with Kinetic Control (21h); MM-EF = Minimum Medium with End of Fermentation (72h); BA-KC = Basal Medium with Kinetic Control (21h); BA-EF = Basal Medium with End of Fermentation (72h). T0-T20 = Transfer numbers. Species appearing at frequencies below 1% in all samples were omitted.

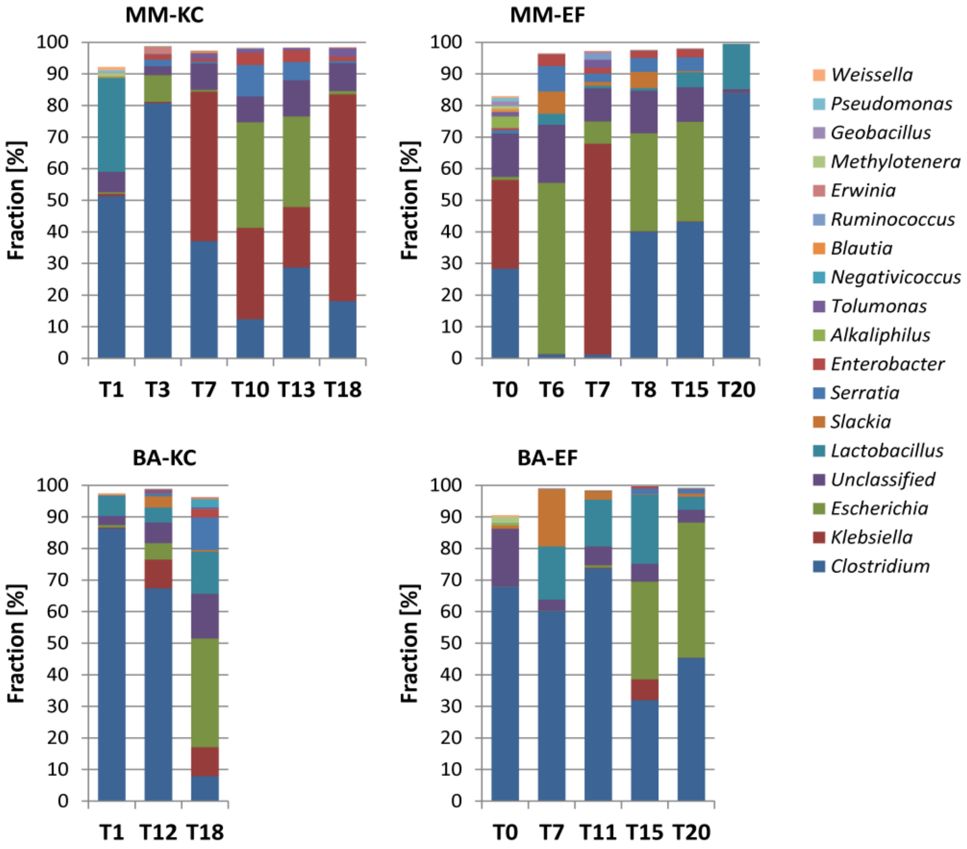

S2. Metagenomic classification of the MMC at the genus level. Results of batch transfers, during the enrichment of activated sludge. MM-KC = Minimum Medium with Kinetic Control (21h); MM-EF = Minimum Medium with End of Fermentation (72h); BA-KC = Basal Medium with Kinetic Control (21h); BA-EF = Basal Medium with End of Fermentation (72h). T0-T20 = Transfer numbers. Species appearing at frequencies below 1% in all samples were omitted.

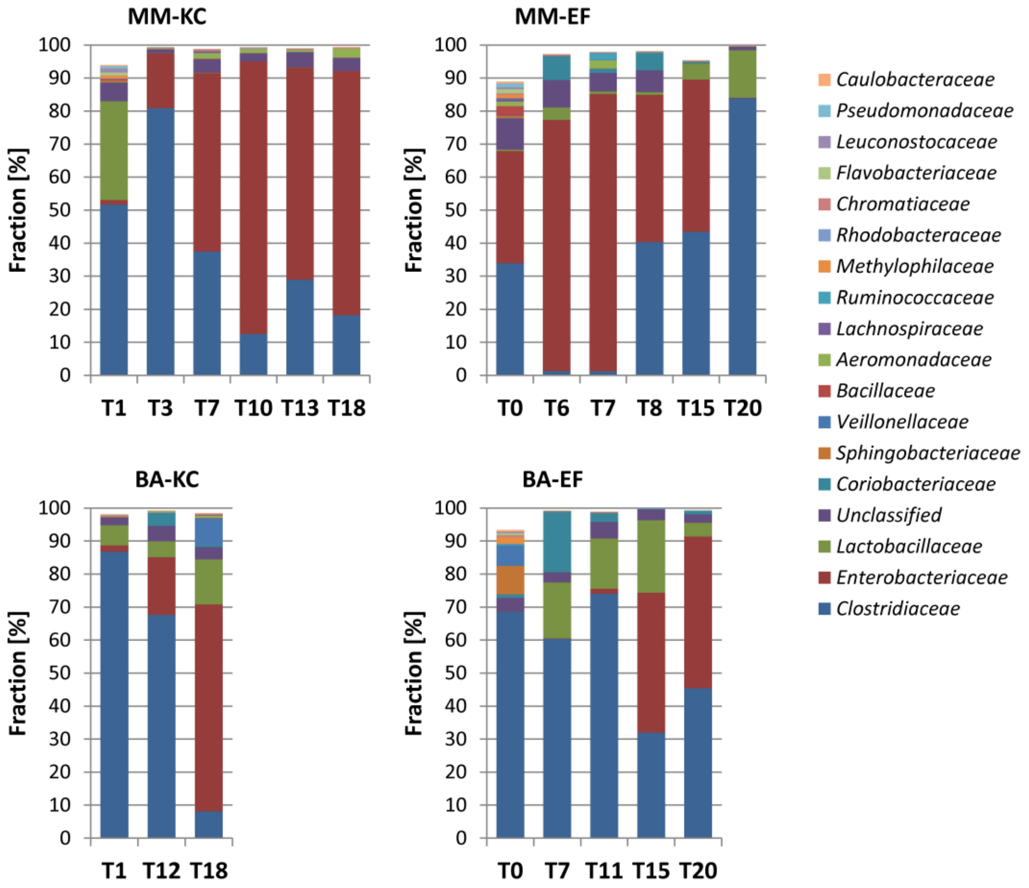

S3. Metagenomic classification of the MMC at the family level. Results of batch transfers, during the enrichment of activated sludge. MM-KC = Minimum Medium with Kinetic Control (21h); MM-EF = Minimum Medium with End of Fermentation (72h); BA-KC = Basal Medium with Kinetic Control (21h); BA-EF = Basal Medium with End of Fermentation (72h). T0-T20 = Transfer numbers. Families appearing at frequencies below 1% in all samples were omitted.

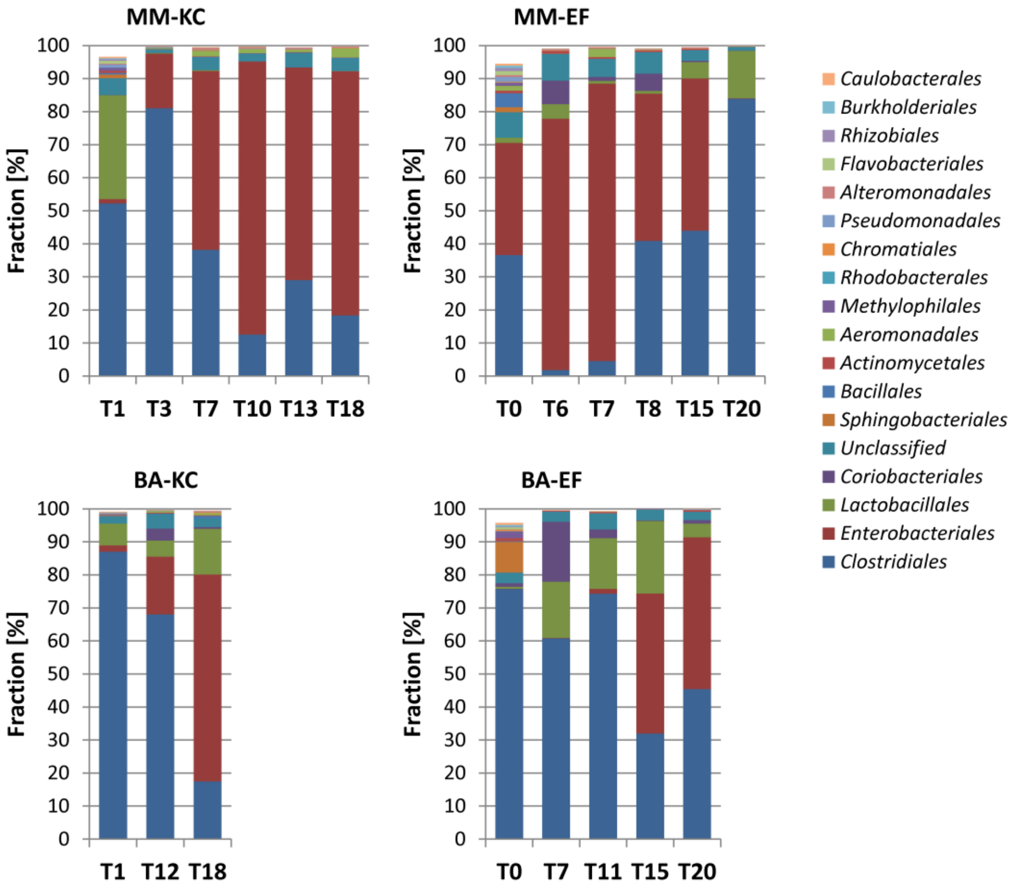

S4. Metagenomic classification of the MMC at the order level. Results of batch transfers, during the enrichment of activated sludge. MM-KC = Minimum Medium with Kinetic Control (21h); MM-EF = Minimum Medium with End of Fermentation (72h); BA-KC = Basal Medium with Kinetic Control (21h); BA-EF = Basal Medium with End of Fermentation (72h). T0-T20 = Transfer numbers. Orders appearing at frequencies below 1% in all samples were omitted.

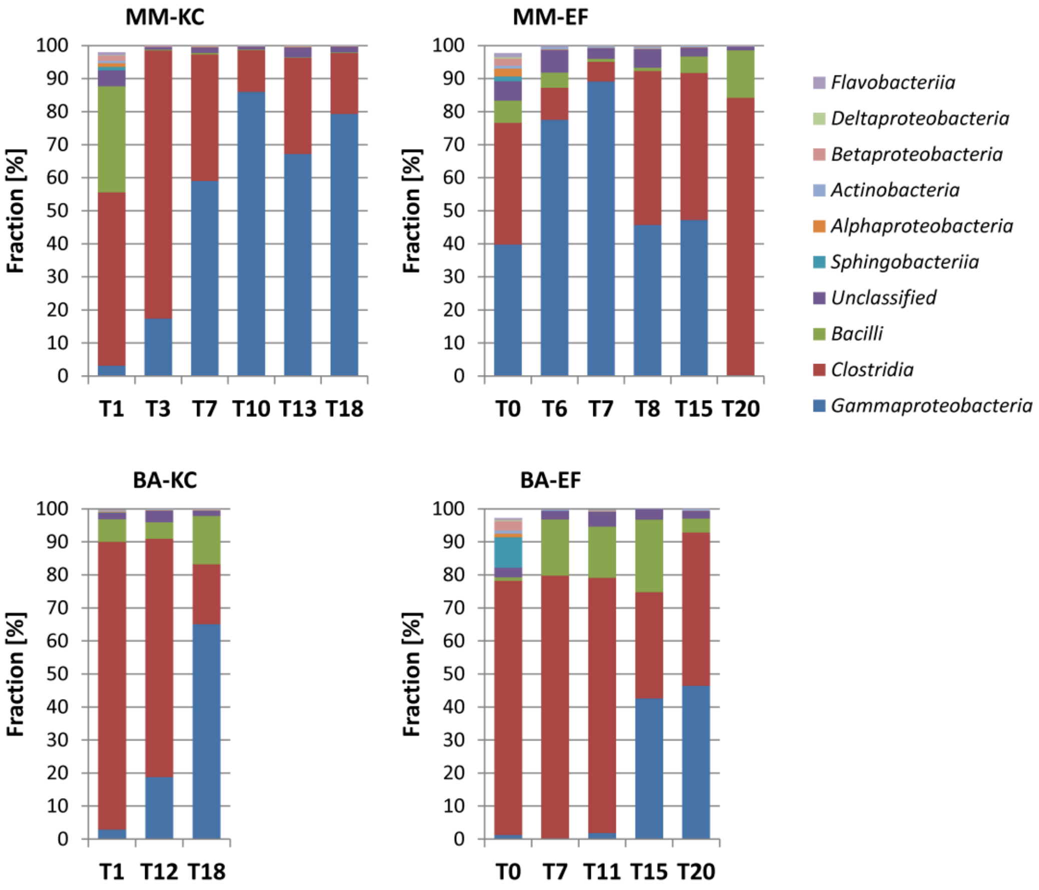

S5. Metagenomic classification of the MMC at the class level. Results of batch transfers, during the enrichment of activated sludge. MM-KC = Minimum Medium with Kinetic Control (21h); MM-EF = Minimum Medium with End of Fermentation (72h); BA-KC = Basal Medium with Kinetic Control (21h); BA-EF = Basal Medium with End of Fermentation (72h). T0-T20 = Transfer numbers. Classes appearing at frequencies below 1% in all samples were omitted.

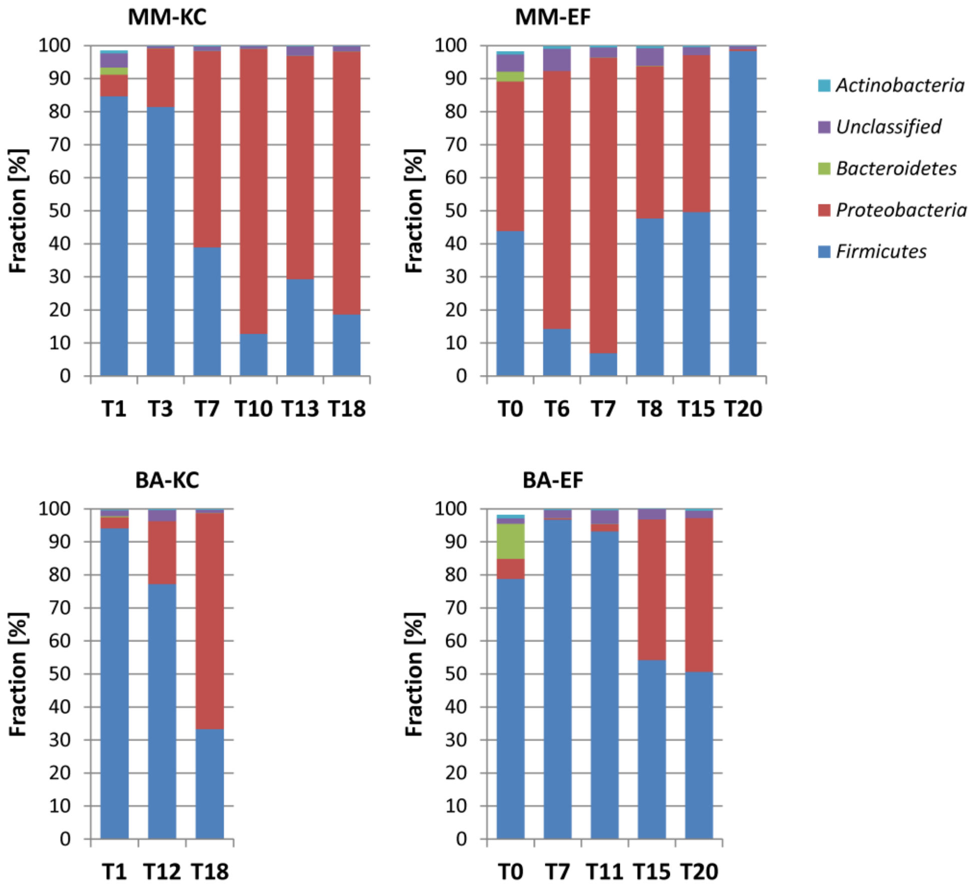

S6. Metagenomic classification of the MMC at the phylum level. Results of batch transfers, during the enrichment of activated sludge. MM-KC = Minimum Medium with Kinetic Control (21h); MM-EF = Minimum Medium with End of Fermentation (72h); BA-KC = Basal Medium with Kinetic Control (21h); BA-EF = Basal Medium with End of Fermentation (72h). T0-T20 = Transfer numbers. Phyla appearing at frequencies below 1% in all samples were omitted.

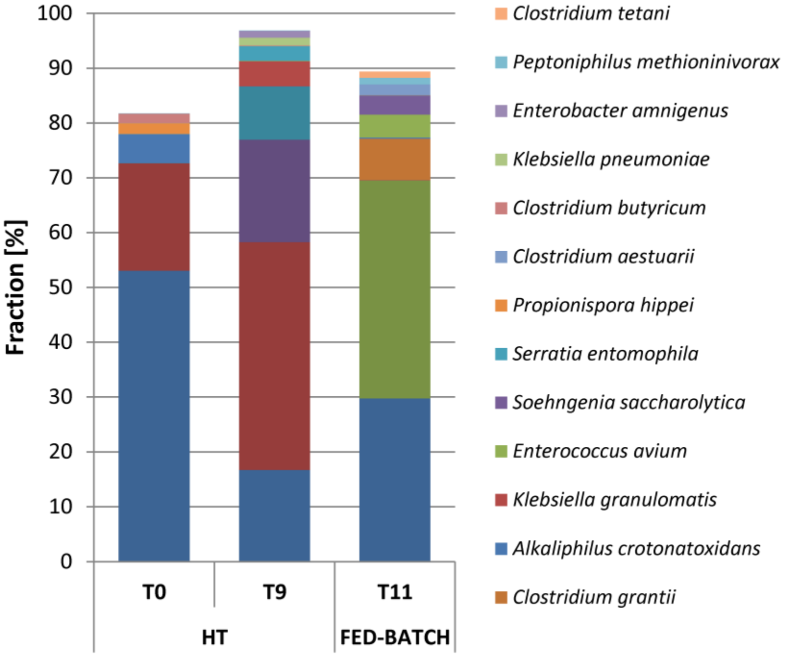

S7. Metagenomic classification of the MMC, at the species level, for the anaerobic sludge enriched on hexane-pretreated crude glycerol in batch tests (HT) and with the untreated crude glycerol in fed-batch. T0-T11= Transfer numbers. Species appearing at frequencies below 1% in all samples were omitted.

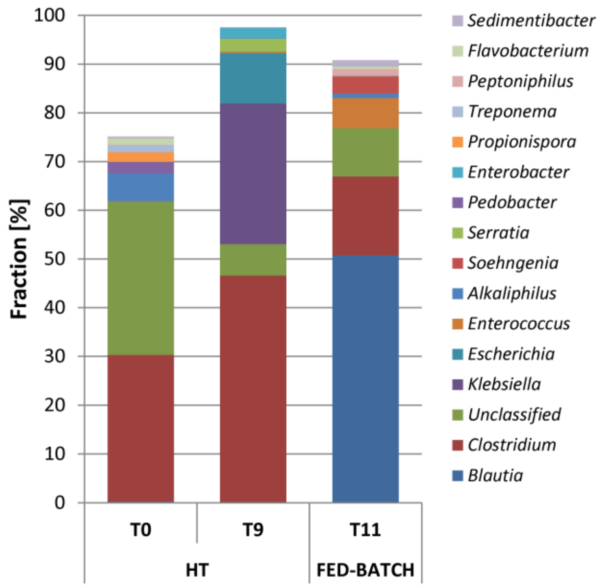

S8. Metagenomic classification of the MMC, at the genus level, for the anaerobic sludge enriched on hexane-pretreated crude glycerol in batch tests (HT) and with the untreated crude glycerol in fed-batch. T0-T11= Transfer numbers. Species appearing at frequencies below 1% in all samples were omitted.

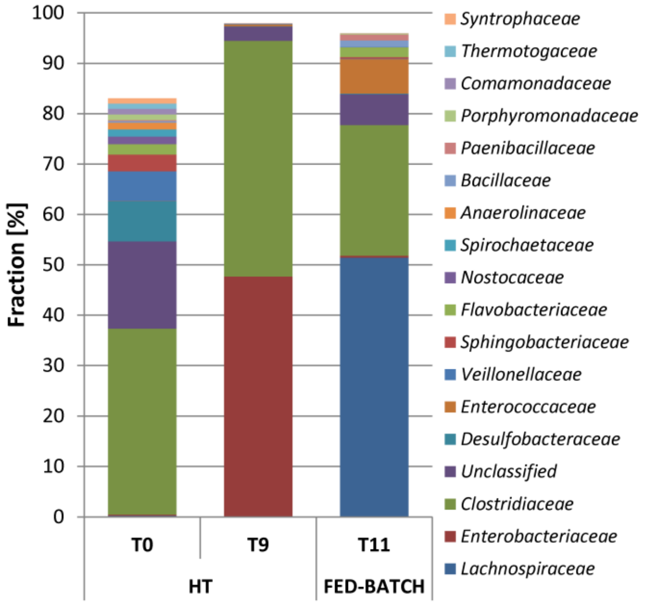

S9. Metagenomic classification of the MMC, at the family level, for the anaerobic sludge enriched on hexane-pretreated crude glycerol in batch tests (HT) and with the untreated crude glycerol in fed-batch. T0-T11= Transfer numbers. Families appearing at frequencies below 1% in all samples were omitted.

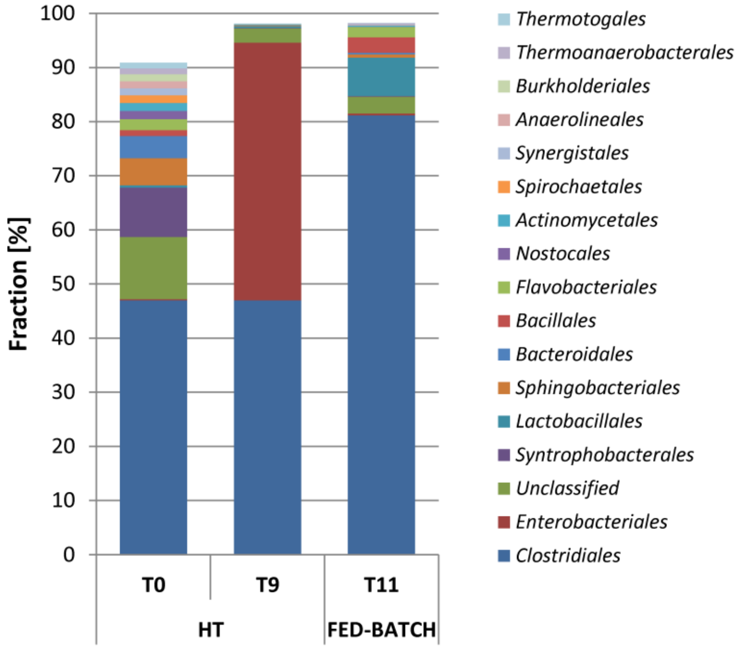

S10. Metagenomic classification of the MMC, at the order level, for the anaerobic sludge enriched on hexane-pretreated crude glycerol in batch tests (HT) and with the untreated crude glycerol in fed-batch. T0-T11= Transfer numbers. Orders appearing at frequencies below 1% in all samples were omitted.

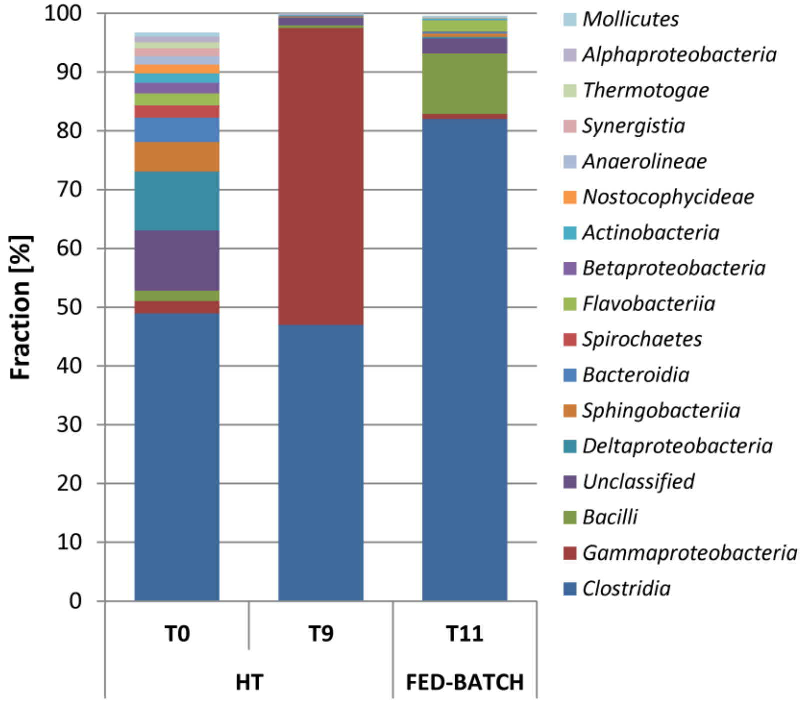

S11. Metagenomic classification of the MMC, at the class level, for the anaerobic sludge enriched on hexane-pretreated crude glycerol in batch tests (HT) and with the untreated crude glycerol in fed-batch. T0-T11= Transfer numbers. Classes appearing at frequencies below 1% in all samples were omitted.

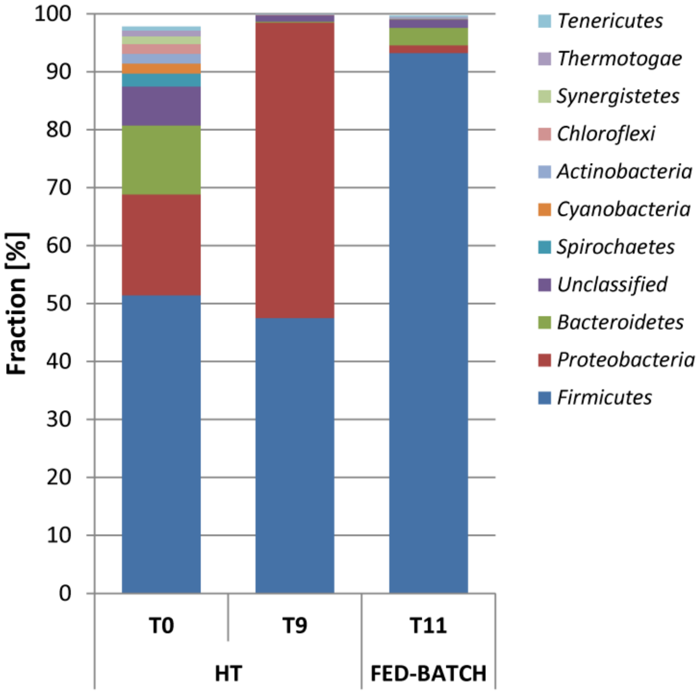

S12. Metagenomic classification of the MMC, at the phylum level, for the anaerobic sludge enriched on hexane-pretreated crude glycerol in batch tests (HT) and with the untreated crude glycerol in fed-batch. T0-T11= Transfer numbers. Phyla appearing at frequencies below 1% in all samples were omitted.
